# Previous History of American Tegumentary Leishmaniasis Alters Susceptibility and Immune Response Against *Schistosoma mansoni* Infection in Humans

**DOI:** 10.3389/fimmu.2021.630934

**Published:** 2021-03-11

**Authors:** Guilherme Silva Miranda, Samira Diniz Resende, Diogo Tavares Cardoso, Genil Mororó Araújo Camelo, Jeferson Kelvin Alves Oliveira Silva, Vanessa Normandio de Castro, Stefan Michael Geiger, Mariângela Carneiro, Deborah Negrão-Corrêa

**Affiliations:** ^1^Laboratory of Immunohelminthology and Schistosomiasis, Department of Parasitology, Institute of Biological Sciences, Federal University of Minas Gerais, Belo Horizonte, Brazil; ^2^Laboratory of Biology, Department of Biology, Institute of Education, Science and Technology of Maranhão, São Raimundo das Mangabeiras, Brazil; ^3^Laboratory of Intestinal Helminthiasis, Department of Parasitology, Institute of Biological Sciences, Federal University of Minas Gerais, Belo Horizonte, Brazil; ^4^Laboratory of Epidemiology of Infectious and Parasitic Diseases, Department of Parasitology, Institute of Biological Sciences, Federal University of Minas Gerais, Belo Horizonte, Brazil

**Keywords:** schistosomiasis, tegumentary leishmaniasis, human population, susceptibility, immune response

## Abstract

Schistosomiasis and Leishmaniasis are chronic parasitic diseases with high prevalence in some tropical regions and, due to their wide distribution, a risk of co-infections is present in some areas. Nevertheless, the impact of this interaction on human populations is still poorly understood. Thus, the current study evaluated the effect of previous American Tegumentary Leishmaniasis (ATL) on the susceptibility and immune response to *Schistosoma mansoni* infection in residents from a rural community in Northern of Minas Gerais state, Brazil, an area endemic for both parasitic infections. The participants answered a socioeconomic questionnaire and provided stool and blood samples for parasitological and immunological evaluations. Stool samples were examined by a combination of parasitological techniques to identify helminth infections, especially *S. mansoni* eggs. Blood samples were used for hemograms and to measure the serum levels of cytokines and chemokines. Reports on previous ATL were obtained through interviews, clinical evaluation forms, and medical records. *S. mansoni* infection was the most prevalent parasitic infection in the study population (46%), and the majority of the infected individuals had a very low parasite burden. In the same population, 93 individuals (36.2%) reported previous ATL, and the prevalence of *S. mansoni* infection among these individuals was significantly higher than among individuals with no ATL history. A multiple logistic regression model revealed that *S. mansoni* infection was positively associated with higher levels of CCL3 and CCL17, and a higher frequency of IL-17 responders. Moreover, this model demonstrated that individuals with an ATL history had a 2-fold higher probability to be infected with *S. mansoni* (OR = 2.0; 95% CI 1.04–3.68). Among *S. mansoni*-infected individuals, the logistic regression demonstrated that a previous ATL history was negatively associated with the frequency of IL-17 responders and CXCL10 higher responders, but positively associated with higher IL-27 responders. Altogether, our data suggest that previous ATL may alter the susceptibility and the immune response in *S. mansoni*-infected individuals, which may likely affect the outcome of schistosomiasis and the severity of the disease in humans.

## Introduction

Schistosomiasis is one of the most important neglected tropical diseases (NTDs) in the world and causes significant morbidity and mortality in human populations (1). Globally, it is estimated that the disease affects more than 240 million people in 78 countries, located mainly in Africa, Latin America, the Middle East, and Southeast Asia (2, 3). The severity of schistosomiasis is related to the parasite species, parasite burden, the host's nutritional status, the frequency of reinfections, exposure time, type and intensity of the host's immune response, and comorbidities (4–8).

Experimental infections with *S. mansoni* in mice (9) and epidemiological studies with infected individuals (5, 10, 11) have demonstrated that a predominantly Th1 type immune response is induced during migration and maturation of the parasite. This initial stage is marked by increased production of pro-inflammatory cytokines, such as interleukin-1 (IL-1), IL-2, IL-6, IL-12, tumor necrosis factor-alpha (TNF-α), and interferon-gamma (IFN-γ) (10–15). After oviposition, soluble parasite egg antigens stimulate the differentiation of a Th2 type immune response, which is mainly characterized by the production of the cytokines IL-4, IL-5, and IL-13, and outweighs the initial type 1 response (9, 15–17). The type 2 immune response is also responsible for inducing a granulomatous cellular response, rich in eosinophils, macrophages, and fibroblasts around the parasite eggs retained in the host tissues (known as granuloma), which can progressively trigger fibrosis, especially in the intestine and the hepatic portal system (18–20). In parallel, the infection also stimulates the production of IL-10 and other regulatory responses, which participate in granuloma modulation during chronic schistosomiasis and promotes a less severe pathology in the majority of infected individuals (21, 22). Granuloma formation and its modulation are key processes for the morbidity of schistosomiasis. Although the granulomatous response promotes an intense inflammatory reaction and can generate severe fibrosis in the vertebrate host, its formation also represents an important host defense mechanism, since the cellular reaction allows the containment of tissue damage caused by egg antigens (9, 11, 20). However, several situations that alter the immune balance, such as co-infections, may change the outcome and morbidity of schistosomiasis.

Like schistosomiasis, American Tegumentary Leishmaniasis (ATL) is a parasitic disease widely distributed in tropical and subtropical countries, mainly in the Americas, Mediterranean, and Asia, and presents approximately 0.7–1.2 million cases per year (23). Experimental studies indicated that the control of intracellular *Leishmania* sp. is mainly associated with the development of a Th1 immune response, in which a higher IFN-γ and TNF-α production increases the levels of nitric oxide (NO) and reactive oxygen species (ROS), thus destroying the amastigote stages (24–29). However, while an early type 1 response was shown to be essential to control the parasite load, an exacerbated response can cause severe tissue damage (25, 29–32). In leishmaniasis, the induction of a Th2 response, with increased IL-4, IL-5 and/or IL-13 production, and regulatory responses, via the production of IL-10 and/or transforming growth factor β (TGF-β), modulate the protective immune response and favor *Leishmania* persistence. On the other hand, such responses were shown to be important to prevent or diminish tissue injuries and facilitate tissue healing (25, 26, 28–31, 33).

Although there are endemic areas in tropical countries where both *Schistosoma* and *Leishmania* are transmitted (34–37), the possible effects of such co-infections on the host susceptibility and/or morbidity have been poorly documented. Experimental models for *S. mansoni* and *L. major* co-infection indicate that the induction of type 2 immunity in response to *S. mansoni* infection reduces the proinflammatory Th1 mechanisms necessary for the effective elimination of *L. major*, which may cause persistent parasitism and a change in the pathology of leishmaniasis, thus delaying the development and resolution of the lesions (38). Moreover, it has been shown that individuals infected with *L. braziliensis* and parasitized by helminths, including *S. mansoni*, present a poor response to treatment, with a consequent delay in cure time (35, 37, 39). Nevertheless, the possible effects of leishmaniasis on the evolution of schistosomiasis in human populations remain mostly unknown. Aiming to address this issue, we evaluated *S. mansoni* susceptibility and the host's immune response in residents of a rural community in an endemic area with transmission of ATL and intestinal schistosomiasis located in the state of Minas Gerais, Brazil. Our data showed that a history of ATL was associated with increased susceptibility to schistosomiasis and modulation of systemic inflammatory parameters in individuals with active *S. mansoni* infection.

## Patients and Methods

### Study Population and Ethical Considerations

The current study is part of a research project that began in 2014 and aimed to characterize schistosomiasis and evaluate the effectiveness of diagnosis methods in individuals from endemic areas showing a low parasite burden (40–42). The study was approved by the Research Ethics Committee (Institute René Rachou/Fiocruz/MG) and all project details have been registered on the Brazilian Platform for Research with Human Subjects (CAAE#21824513.9.0000.5091). All enrolled participants were required to sign an informed consent form and individuals with stool samples positive for parasites received oral treatment at the local health clinic, using Praziquantel (40–60 mg/kg) for *S. mansoni*, albendazole (400 mg, single dose) for intestinal helminths, and metronidazole (250 mg/2 × /5 days) for intestinal protozoans.

**Figure 1:**
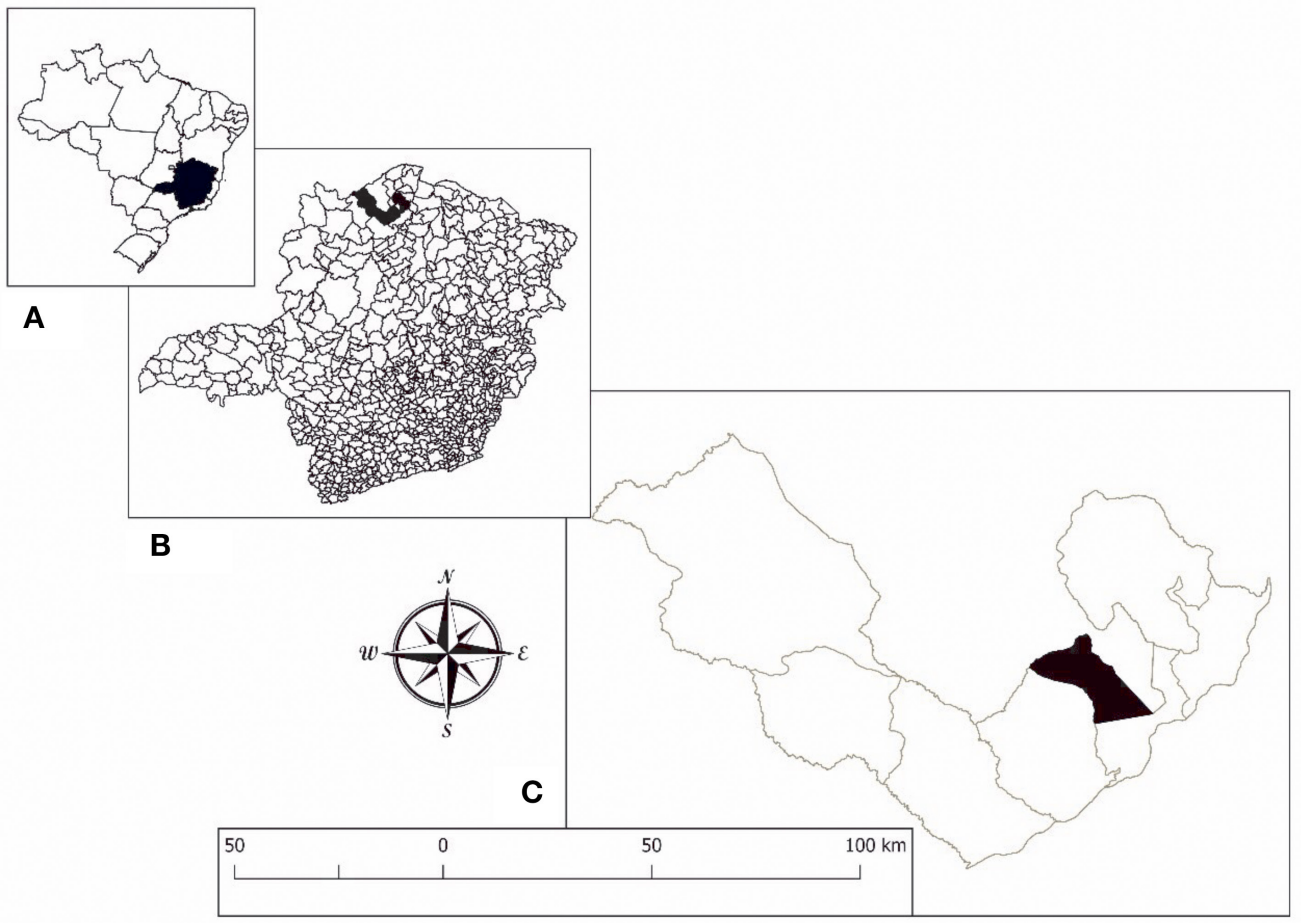
Location of the study area. **(A)** State of Minas Gerais in Brazil. **(B)** Municipality of Januária. **(C)** District of Brejo do Amparo.

**Figure 2:**
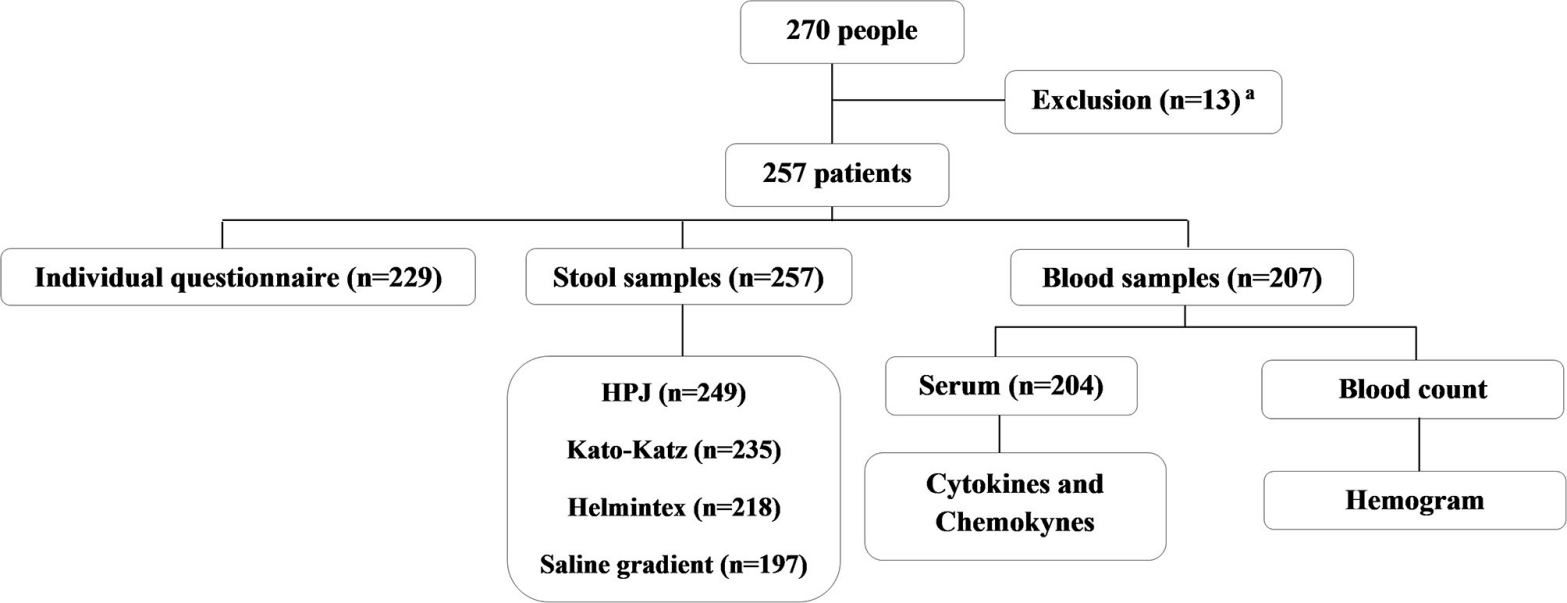
Flowchart describing the endemic population enrolled in the study, the sample collection, and the analyses performed. All the individuals that participated in the study were residents from the rural community of Brejo do Amparo, Januária, Minas Gerais, Brazil. ^a^Residents who did not sign the consent form, did not have the established minimum age allowed for our study, pregnant women and/or individuals with cognitive diseases that would interfere with the obtention of information necessary for the study.

For the current study, we used the cross-sectional population-based data obtained from residents of a rural community in the district of Brejo do Amparo (15°25′54″S 44°24′42″W), Municipality of Januária, located in the Northern region of the state of Minas Gerais (Figure 1), an endemic region for schistosomiasis and ATL (40, 41, 43). At the beginning of the study, the rural community had approximately 270 inhabitants, of which 257, aging from two to 88 years old, agreed to participate in the population-based study. All participants signed the consent form and were invited to collect urine and stool samples for parasitological analyses, as previously detailed by Oliveira et al. (40). Two-hundred and twenty-nine participants or their legal guardians answered a socioeconomic and demographic questionnaire that also contained questions regarding exposure to schistosomiasis and the history of ATL, which is frequent in the area and very well-known to the residents. The questionnaires were pre-coded and applied by previously trained interviewers. Individuals aging from five to 75 years old were invited to provide a blood sample for the evaluation of immunological parameters, as detailed in Figure 2. Parasitological, immunological, and socioeconomic data obtained from this population-based study (40, 41) were used in the current research.

### Determination of *S. mansoni* Infection and Parasite Burden in the Study Population

*S. mansoni* infection was identified in the study population by a combination of the parasitological tests specified in Figure 2 and detailed in Oliveira et al. (40), and the results were integrated into the database of the present study.

In brief, each participant was asked to collect fecal samples on three consecutive days. All the samples were taken to the field laboratory in Januária, homogenized, and immediately processed. The first fecal sample was initially processed and used to perform HPJ (Hoffman, Pons and Janer) technique (44) and 14 Kato-Katz slides (45, 46). From the same fecal sample, 500 mg were used to perform saline gradient method (47) and 30 g were processed by the Helmintex® technique (48). The second and third fecal samples were used to prepare two Kato-Katz slides per sample. The slides containing fecal material from the different parasitological tests were examined under the microscope by trained technicians to evaluate the presence of helminth eggs and protozoan cysts. *S. mansoni* infection was diagnosed by the presence of the parasite eggs in either one of the several parasitological methods (Kato-Katz, HPJ, saline gradient, Helmintex®). The HPJ, saline gradient, and Helmintex® techniques were used as qualitative tests to identify the presence or absence of *S. mansoni* eggs. The *S. mansoni* burden was estimated as the mean number of eggs obtained from six slides of Kato-Katz (the first two slides from each fecal sample) multiplied by 24 and plotted as the number of *S. mansoni* eggs per gram of feces (epg) (46). Individuals showing *S. mansoni* eggs in the qualitative parasitological tests (Helmintex® and saline gradient) and with no *Schistosoma* eggs in the six Kato-Katz slides (≤ 3 epg) were designated as infected and assigned a parasite burden of 2 epg.

### Hematological and Systemic Immune Response Evaluation

Blood samples were collected in the morning after fasting and using EDTA-containing tubes for a complete blood count performed by a commercial local laboratory and EDTA-free tubes to obtain the serum for immunological analysis, as previously detailed (41, 49). In the current work, data on hemoglobin levels and the number of circulating red blood cells, eosinophils, and platelets were evaluated as the median value and as frequency of altered values among individuals with different infection status. The standard values for red blood cells were 5/mm^3^ for males and 4.5/mm^3^ for females. For hemoglobin concentration, they were 14.9 g/dl for males and 13.2 g/dl for females. Value of red blood cells counts and hemoglobin concentration lower than the standard value were considered altered. Moreover, values below 140.000/mm^3^ for platelets and above 500/mm^3^ for eosinophils were also considered altered (50, 51).

The serum samples were used for the quantification of TNF-α (7.8–1,000 pg/ml), IL-5 (11.7–1500 pg/ml), IL-6 (4.7–600 pg/ml), IL-10 (15.6–2,000 pg/ml), IL-13 (46–6,000 pg/ml), IL-17 (7.8–1,000 pg/ml), IL-27 (78.1–10,000 pg/ml), IL-33 (11.7–1,500 pg/ml), CCL3 (3.9–500 pg/ml), CCL5 (7.8–1,000 pg/ml), CCL11 (7.8–1,000 pg/ml), CCL17 (3.9–500 pg/ml), and CXCL10 (15.6–2,000 pg/ml) by sandwich enzyme-linked immunosorbent assay (ELISA), using commercially available detection kits (R&D Systems, USA) and following the manufacturer's recommendations. Briefly, each serum sample was diluted at 1:2 in phosphate-buffered saline (PBS) with 0.1% of bovine serum albumin (BSA) (Sigma-Aldrich) (w/v) and tested in duplicate. Serum samples used to quantify CCL5 and CCL3 were further diluted 1:20 and 1:3, respectively. Known concentrations of the recombinant proteins were used to generate a standard curve to determine the concentration (pg/ml) of the samples based on OD readings. The absorbance was determined using a 450 nm filter in the ELISA reader (VersaMax, Molecular Devices, Sunnyvale, CA).

Most individuals showed no detectable serum levels of the cytokines TNF-α, IL-5, IL-6, IL-10, IL-13, IL-17, and IL-33, and the data was plotted and analyzed as the frequency of individuals with detectable cytokine (designated as responders) in each group. Serum levels of the chemokines (CCL3, CCL5, CCL11, CCL17, and CXCL10), and the cytokines IL-6 and IL-27 were plotted as continuous values and categorized as high and low responders based on the median concentration of each mediator.

### Evaluation of ATL History

Previous reports of tegumentary leishmaniasis among the study subjects were identified in the individual questionnaires and the clinical files, based on the report of the clinical skin and mucosal manifestations of tegumentary leishmaniasis. Additionally, patients with previous symptomatic ATL were identified in the medical records of the Center for Treatment and Research in Leishmaniasis of Januária (CTPLJ), a reference health facility for the treatment of the disease in the region. Information regarding leishmaniasis status registered in the medical records was added to the database. Access to information from the medical records on previous *Leishmania* infections and their use for research purposes were allowed by the patients through written informed consent and approved by the Ethics Committee of the Federal University of Minas Gerais (CAAE: 69370517.3.0000.5149).

### Statistical Analysis

The statistical analyses were performed in the STATA version 11.1 software (Stata Corp., College Station, TX, USA) and the graphs were constructed using the software GraphPad-Prism 6 (Prism Software, Irvine, California, USA).

The Shapiro-Wilk test was used to verify the normality of the data. For non-parametric data the medians were compared by the Kruskal-Wallis test followed by the Dunn's test. Categorical variables were compared by the chi-square statistical test. Values of *p* < 0.05 were considered statistically significant.

Initially, the first logistic regression model was performed to assess the possible associations between *S. mansoni* infection (response variable) and the analyzed covariates, including demographics parameters, report of previous ATL, hematological and immunological response that were selected based on the univariate analysis. The second model was performed to better understand the effect of ATL history on schistosomiasis, and for this purpose, only the *S. mansoni*-infected individuals were included. For this second model the response variable was previous ATL in *S. mansoni* infected population (SM+ATL) and the analysis aimed to investigated possible associations between the covariates (hematological and immunological parameters) and the response variable (SM+ATL) in the *S. mansoni*-infected population. Variables with a *p*-value < 0.25 in the univariate analysis were selected to compose the multiple logistic regression models. The step-by-step backward method was used, and the final variables that remained in the models were those that presented a significance level of *p* < 0.05 after adjusted to the others. Age was used to adjust the models. The strength of association was determined by OR with 95% CI.

## Results

### Characterization of the Study Population

As previously shown (40, 41), 257 residents from a rural area of Brejo do Amparo participated in the study, 122 (46.7%) of which were males and 135 (53.3%) females, with a median age of 32 years old (interquartile range: 15–51). According to the socioeconomic questionnaires answered by 229 participants, almost 60% of the individuals had no formal education and 36% lived with less than one Brazilian minimum wage (Supplementary Table 1). *S. mansoni* infection was identified in 118 participants (46%). In addition, 23 individuals (9%) presented hookworm and six (2%) showed *Enterobius vermicularis* eggs in the stool samples, most of which were co-infected by *S. mansoni*. There was also one case of *Trichuris trichiura* (0.4%) and one case of *Strongyloides stercoralis* (0.4%) infection. Among intestinal protozoan parasites, only *Giardia lamblia* (*n* = 4; 1.5%) and *Entamoeba histolytica*/*dispar* (*n* = 9; 3.5%) were found (Supplementary Table 1).

### *S. mansoni* Parasite Burden

In the study population, *S. mansoni*-infected individuals presented a median of 4 epg (interquartile range: 2–20 epg), confirming the low parasite burden of the population. It was verified that 108 individuals (91.5% of the infected population) eliminated <100 epg, while only 10 infected individuals (8.5%) eliminated 100 or more epg, which represents moderate to heavy intensity infection, according to the World Health Organization (WHO) (52) (Supplementary Table 2). No statistically significant differences were observed in the number of *S. mansoni* eggs eliminated by infected male and female individuals or among the different age groups evaluated in this population (Supplementary Table 2).

### Characterization of Tegumentary Leishmaniasis History in the Study Population

Using the information obtained from the individual questionnaires, clinical files, and the medical records of individuals who attended the CTPLJ, 93 subjects (36.2%) had reports of previous cases of ATL and were considered for the proposed analyzes (Table 1).

**Table 1:** Social aspects and clinical characteristics of individuals with a previous report of American Tegumentary Leishmaniasis (ATL) living in the rural community of Brejo do Amparo, Minas Gerais, Brazil.

**SOCIAL ASPECTS (*n* = 93)**	
**Gender**	***N* (%)**
Male	40 (43)
Female	53 (57)
**Age**	
≤ 10	7 (7.5)
11-20	16 (17.2)
21-40	27 (29)
41-60	31 (33.3)
>60	12 (13)
**CLINICAL ASPECTS (*n* = 41)**	
**N^°^ of lesions (*n* = 41)**	*N* (%)
One	33 (80.5)
>One	08 (19.5)
**Clinical form (*n* = 41)**	
Cutaneous	40 (97.6)
Mucosal	01 (2.4)
**No of affected members (*n* = 41)**	
One	38 (92.7)
>One	3 (7.3)
**Treatment time (*n* = 38)^a^**	
<30 days	06 (15.8)
>30 days	32 (84.2)
**Relapse/Reinfection (*n* = 41)**	
No	32 (78)
Yes	9 (22)

a *Three patients had no data for this item*.

Only 41 (44%) of the 93 individuals who reported ATL had medical records at the CTPLJ (2010–2015), and 38 of them had classical clinical manifestations of tegumentary leishmaniasis in the past 5 years before this study. For these individuals, leishmaniasis was confirmed by specific tests, mainly biopsy and/or Montenegro's intradermal test, and clinical and therapeutic aspects related to the infection were obtained. The cutaneous form of ATL (*n* = 40; 97.6%) with a single lesion (80.5%) found in one limb (92.7%) was predominant among these subjects. Moreover, the Glucantime treatment for leishmaniasis in the individuals evaluated at the CTPLJ (44% of the previous ATL cases), took more than 30 days to show an effect in about 80% of the cases, and relapses/reinfections were reported in approximately 22% of the individuals (Table 1).

### The Effect of Previous ATL on *S. mansoni* Infection

The prevalence of *S. mansoni* infection was higher among individuals with an ATL history when compared with individuals with no reported ATL (43.2 vs. 30.2%, respectively; *p* = 0.032) (Table 2). Interestingly, although no difference was observed between the number of *S. mansoni* eggs eliminated in the feces of individuals with or without an ATL history, all patients with moderate to heavy *S. mansoni* infection (*n* = 10) had no report of previous ATL. Therefore, the frequency of individuals with intense parasite burden was higher among infected individuals without previous ATL (*p* = 0.004) (Table 2).

**Table 2:** Parasite burden and prevalence of *S. mansoni* infection and report of ATL history in individuals living in the rural community of Brejo do Amparo, Januária, Minas Gerais, Brazil.

	**Prevalence of** ***S. mansoni***			
		**Positive (%)**	**Negative (%)**	***P***
ATL history	Positive (%)	51 (43.2)	42 (30.2)	0.032^*^
	Negative (%)	67 (56.8)	97 (69.8)	
	**Total**	118	139	
	***S. mansoni*** **burden**^**a**^			
		**≤** **99 epg (low %)**	**>** **99 epg (moderate/heavy %**)	***P***
*S. mansoni*		57 (52.8)	10 (100)	0.004^*^
*S. mansoni* + ATL history		51 (47.2)	0 (0)	

* *Significant statistical difference (p < 0.05) based on the chi-square test*.

a *According to the WHO (52)*.

### Hematological Parameters

Blood samples were collected from 207 individuals of the study population for the measurement of hematological parameters. No significant differences were observed in the median levels of hemoglobin and the number of red blood cells, platelets, and eosinophils among infected or non-infected individuals, with or without an ATL history (data not shown). Moreover, about 9% of the study population showed low levels of hemoglobin, 3% had red blood cell counts below the normal, and 5% had altered counts of platelets, albeit with no differences in the frequencies among the groups (Table 3). However, the frequency of eosinophilia (>500/mm^3^) was significantly higher in individuals infected with *S. mansoni* only (51%; *n* = 29; *p* = 0.022), defined as SM group, when compared with the rest of the population (Table 3).

**Table 3:** Frequency of normal and altered values for hemoglobin, red blood cells, platelets, and eosinophils in individuals with different parasitic status living in the rural community of Brejo do Amparo, Januária, Minas Gerais, Brazil.

	**Control**	**SM**	**ATL**	**SM+ATL**	***p*^a^**	***p*^b^**
	***N* = 67 (32.0%)**	***N* = 57 (28.0%)**	***N* = 36 (17.0%)**	***N* = 47 (23.0%)**		
**Hemoglobin (*****n*** **=** **207)**						
Normal	59 (88.0)	53 (93.0)	32 (89.0)	44 (94.0)	0.674	0.898
Altered	8 (22.0)	4 (7.0)	4 (11.0)	3 (6.0)		
**Red blood cell (*****n*** **=** **207)**						
Normal	65 (97.0)	56 (98.0)	33 (92.0)	47 (100)	0.142	0.362
Altered	2 (3.0)	1 (2.0)	3 (8.0)	0 (0.0)		
**Platelet (*****n*** **=** **207)**						
Normal	66 (98.5)	52 (91.0)	34 (94.5)	45 (96.0)	0.305	0.373
Altered	1 (1.5)	5 (9.0)	2 (5.5)	2 (4.0)		
**Eosinophilia (*****n*** **=** **207)**						
Absent	41 (61.0)	28 (49.0)	29 (81.0)	31 (66.0)	0.022^*^	0.085
Present	26 (39.0)	29 (51.0)	7 (19.0)	16 (34.0)		

*Control, no S. mansoni infection or ATL history; SM, S. mansoni-infected only; ATL, ATL history only; SM+ATL, S. mansoni infection and ATL history; p-value obtained by chi-squared test*;

a *Among all the groups*;

b *Between SM and SM+ATL groups*;

* *Statistically significant (p < 0.05)*.

### Immunological Parameters

Serum samples were collected from 204 individuals of the study population and allowed the measurement of the majority of the circulating immune mediators evaluated in this study. Among these subjects, 54 had *S. mansoni* infection with no ATL history (SM group); 37 reported previous ATL, but no *S. mansoni* eggs found in feces (ATL group); 44 were infected with *S. mansoni* and had an ATL history (SM+ATL group); and the remaining 69 individuals presented with neither active *S. mansoni* infection nor reported ATL (control group). The median age (and 25–75% interquartile range) of the control, SM, ATL and, SM + ATL groups were, respectively, 31 (17–50.25), 30 (15.5–52.5), 34 (21, 5–46) and 40.5 (19.75–50.75). Regarding gender, the percentage of women varied between 46.4–59.5% between groups. However, none of these parameters differed significantly between groups.

All individuals had detectable levels of the cytokines IL-6 and IL-27, and the chemokines CCL3, CCL5, CXCL10, CCL11, and CCL17. No significant difference was detected in the serum levels of IL-6, CCL5, and CXCL10 among the analyzed groups (Table 4). Although the serum concentrations of IL-27 and CCL11 were also statistically similar among the groups, their *p*-values were lower than 0.25; therefore, these mediators were included in the multiple analysis. The serum concentrations of CCL3 and CCL17 were significantly lower in individuals from the ATL group when compared with the SM+ATL and SM groups, respectively (Table 4).

**Table 4:** Serum levels of IL-6, IL-27, CCL3, CCL5, CXCL10, CCL11, and CCL17 in patients with different parasitic infection profiles living in the rural community of Brejo do Amparo, Januária, Minas Gerais, Brazil.

	**Control (*n* = 69)**	**SM (*n* = 54)**	**ATL (*n* = 37)**	**SM+ATL (*n* = 44)**	
	**M_**d**_ (IR 25–75%)**	**M_**d**_ (IR 25–75%)**	**M_**d**_ (IR 25–75%)**	**M_**d**_ (IR 25–75%)**	***p*^**a**^**
IL-6	2.0 (0–6.0)	4.0 (1.0–8.0)	3.0 (0–7.0)	4.0 (1.5–6.5)	0.595
IL-27	492.0 (258.5–759.8)	319.0 (154.0–625.0)	276.0 (161.5–498.0)	418.0 (174.0–655.5)	0.075
CCL3	4,870 (2,330–13,180)	7,105 (3,360–12,490)	3,360 (1,720–10,270)^**≠**^	6,660 (3,360–22,485)^**≠**^	0.029^*^
CCL5	11,783 (6,844–28,854)	11,746 (6,952–22,579)	11,246 (7,672–18,082)	11,465 (6,404–24,130)	0.954
CXCL10	121.0 (68.8–175.5)	127.5 (77.5–194.5)	110.0 (83.5–167.0)	105.5 (69.3–152.5)	0.546
CCL11	92.0 (27.5–260)	59.0 (26.0–192.0)	45.0 (9.0–127.0)	102.0 (33.0–188.0)	0.135
CCL17	207.0 (128.3–477.5)	341.0 (190.5–2,206)^**≠**^	150.0 (57.5–473.5)^**≠**^	233.5 (110.5–1,148)	0.038^*^

*Control, no S. mansoni infection or ATL history; SM, S. mansoni-infected only; ATL, ATL history only; SM+ATL, S. mansoni infection and ATL history; M_d_, median; IR, interquartile range; Unit used: pg/ml*.

a p-value obtained by Kruskal-Wallis, followed by Dunn's multiple comparisons post-test between the groups;

* *Statistically significant (p < 0.05)*.

≠ *Groups of patients that presented statistically significant differences between them*.

Additionally, the frequency of individuals classified as high responders for these mediators (showing serum concentrations higher than the median of the study population) was evaluated in each group (Table 5). The frequencies of high responders for IL-6 and CCL5 were similar among the groups. The frequencies of CXCL10 and CCL11 high responders were also statistically similar in the different groups, but their *p*-values were lower than 0.25 and, therefore, these mediators were also included in the multiple analysis. Interestingly, an increased frequency of CCL17 high responders (>245 pg/ml) in patients from the SM group (almost 70% of the individuals) was detected when compared to the rest of the population (*p* = 0.012). In addition, a statistically higher frequency of CCL17 high responders was observed in the SM group when compared with the SM+ATL group (*p* = 0.034). On the other hand, around 60% of the individuals infected with *S. mansoni*, with or without previous ATL, were high responders for CCL3 (>5,740 pg/ml). Moreover, the control group showed a significantly higher frequency of IL-27 high responders (>388 pg/ml) when compared with the other groups.

**Table 5:** Frequency of serum samples with detectable levels of TNF-α, IL-17, IL-5, IL-13, IL-33, and IL-10, or with high levels of IL-6, IL-27, CCL3, CCL5, CXCL10, CCL11, and CCL17 from individuals with different parasitic infection profiles living in the rural community of Brejo do Amparo, Januária, Minas Gerais, Brazil.

	**Control *N* = 69 (34.0%)**	**SM *N* = 54 (26.5%)**	**ATL *N* = 37 (18.0%)**	**SM+ATL *N* = 44 (21.5%)**	***p*^a^**	***p*^b^**
**TNF-α** **(*****n*** **=** **199)**						
Undetectable	61 (92.0)	45 (86.5)	36 (97.3)	41 (93.2)	0.308	0.288
Detectable	05 (8.0)	07 (13.5)	01 (2.7)	03 (6.8)		
**IL-6 (*****n*** **=** **198)**						
≤ 3pg/ml	39 (59.0)	24 (45.0)	20 (54.0)	20 (47.6)	0.445	0.821
>3pg/ml	27 (41.0)	29 (55.0)	17 (46.0)	22 (52.4)		
**IL-27 (*****n*** **=** **201)**						
≤ 388pg/ml	27 (40.0)	31 (58.5)	25 (68.0)	19 (43.0)	0.024^*^	0.133
>388pg/ml	40 (60.0)	22 (41.5)	12 (32.0)	25 (57.0)		
**IL-17 (*****n*** **=** **201)**						
Undetectable	65 (97.0)	44 (81.5)	36 (100)	43 (98.0)	0.001^*^	0.011^*^
Detectable	02 (3.0)	10 (18.5)	0 (0.0)	01 (2.0)		
**IL-5 (*****n*** **=** **179)**						
Undetectable	56 (93.0)	43 (88.0)	27 (82.0)	32 (86.5)	0.401	0.862
Detectable	04 (7.0)	06 (12.0)	06 (18.0)	05 (13.5)		
**IL-13 (*****n*** **=** **175)**						
Undetectable	34 (54.0)	23 (49.0)	19 (63.0)	19 (54.0)	0.674	0.632
Detectable	29 (46.0)	24 (51.0)	11 (37.0)	16 (46.0)		
**IL-33 (*****n*** **=** **182)**						
Undetectable	36 (56.0)	27 (60.0)	21 (60.0)	28 (74.0)	0.361	0.189
Detectable	28 (44.0)	18 (40.0)	14 (40.0)	10 (26.0)		
**IL-10 (*****n*** **=** **201)**						
Undetectable	49 (73.0)	48 (91.0)	32 (86.5)	41 (93.0)	0.013^*^	0.641
Detectable	18 (27.0)	05 (9.0)	05 (13.5)	03 (7.0)		
**CCL3 (*****n*** **=** **199)**						
≤ 5,740pg/ml	39 (57.0)	22 (41.0)	22 (63.0)	17 (40.5)	0.067	0.979
>5,740pg/ml	29 (43.0)	32 (59.0)	13 (37.0)	25 (59.5)		
**CCL5 (*****n*** **=** **199)**						
≤ 11,696pg/ml	34 (49.0)	26 (48.0)	17 (50.0)	22 (52.0)	0.981	0.681
>11,696pg/ml	35 (51.0)	28 (52.0)	17 (50.0)	20 (48.0)		
**CXCL10 (*****n*** **=** **202)**						
≤ 115pg/ml	32 (48.0)	24 (44.5)	19 (51.0)	27 (61.0)	0.379	0.095
>115pg/ml	35 (52.0)	30 (55.5)	18 (49.0)	17 (39.0)		
**CCL11 (*****n*** **=** **179)**						
≤ 73.5pg/ml	24 (41.0)	29 (62.0)	19 (54.0)	17 (44.0)	0.158	0.094
>73.5pg/ml	34 (59.0)	18 (38.0)	16 (46.0)	22 (56.0)		
**CCL17 (*****n*** **=** **191)**						
≤ 245pg/ml	35 (57.0)	16 (31.0)	22 (61.0)	22 (52.0)	0.012^*^	0.034^*^
>245pg/ml	26 (43.0)	36 (69.0)	14 (39.0)	20 (48.0)		

*Control, no S. mansoni infection or ATL history; SM, S. mansoni-infected only; ATL, ATL history only; SM+ATL, S. mansoni infection and ATL history; p-value obtained by chi-square test*;

a *Among all the groups*;

b *Between SM and SM+ATL groups*;

* *Statistically significant (p < 0.05)*.

In contrast, most of the evaluated individuals showed no detectable levels of TNF-α, IL-17, IL-5, IL-13, IL-33, and IL-10 in the serum. Thus, we compared the frequency of individuals responding to these cytokines in each group (Table 5) and verified that the frequencies of responding individuals for serum TNF-α, IL-5, IL-13, and IL-33 were similar in the different groups. Nevertheless, when only the *S. mansoni*-infected population was analyzed, the group of individuals with previous ATL showed lower frequencies of TNF-α, IL-17, CXCL10, IL-33, and CCL-17 responders compared with the SM group. Interestingly, the frequency of individuals with detectable IL-17 serum levels was statistically higher in the SM group when compared with the rest of the population (*p* = 0.001) and with the SM+ATL group (*p* = 0.011). On the other hand, individuals from the control group showed the highest detection frequency of IL-10 (*p* = 0.013) in the serum.

**Figure 3:**
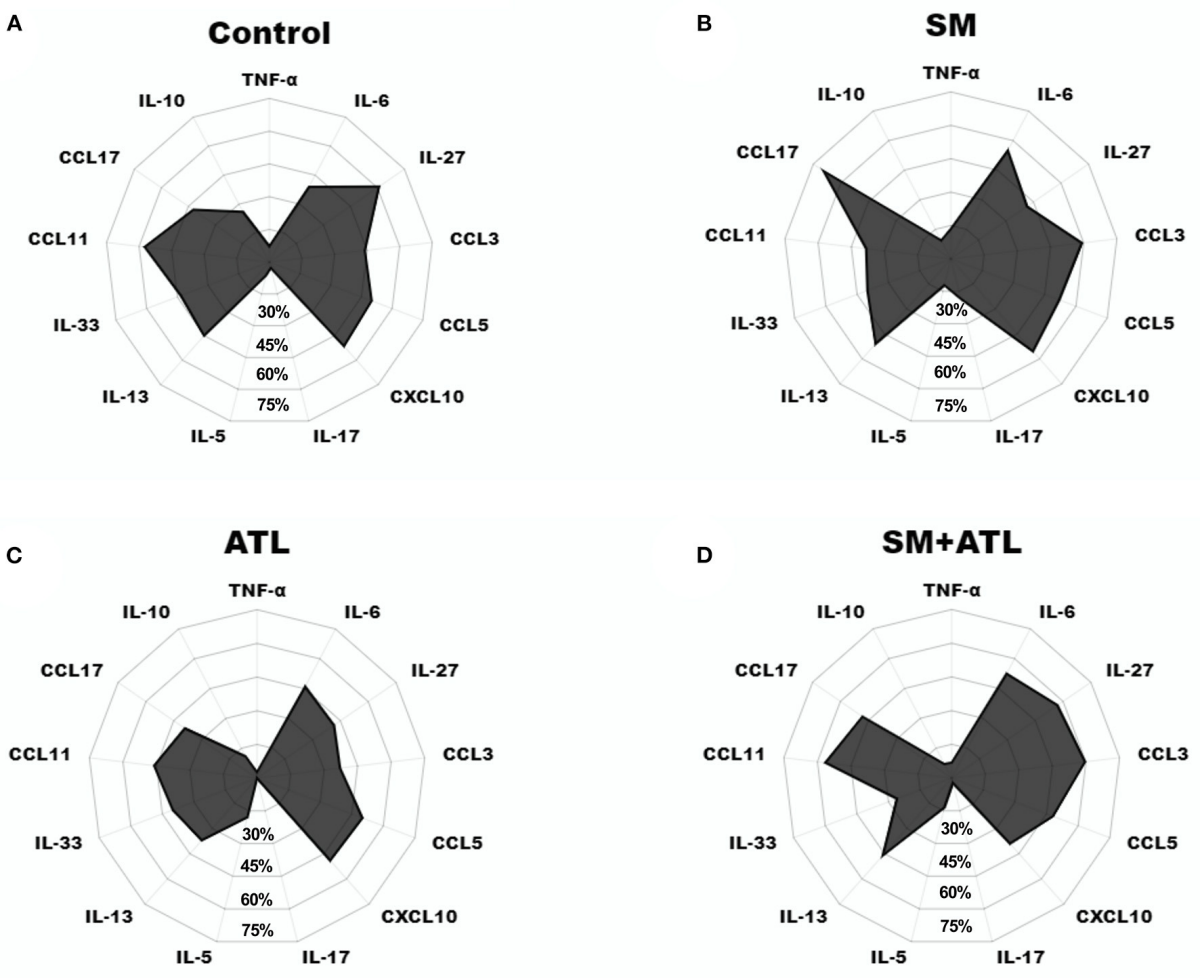
Radar graph showing the frequency of serum samples with detectable levels of TNF-α, IL-17, IL-5, IL-13, IL-33, and IL-10, or with high levels of IL-6, IL-27, CCL3, CCL5, CXCL10, CCL11, CCL17 from individuals with different parasitic infection profiles living in the rural community of Brejo do Amparo, Januária, Minas Gerais, Brazil. **(A)** Control: no *S. mansoni* infection or ATL history; **(B)** SM, *S. mansoni*-infected only; **(C)** ATL, ATL history only; **(D)** SM+ATL, *S. mansoni* infection and ATL history.

The frequencies of responders and high responders for each cytokine and chemokine were comparatively illustrated to show the profile of the immune response in each group of individuals of the study population. The radar graph (Figure 3) clearly illustrates that *S. mansoni* infection increased the frequency of CCL17, IL-6, CCL3, IL-13, CXCL10 and IL-17 responders. However, *S. mansoni*-infected individuals with an ATL history showed a reduction in responders for CCL17, IL-17, CXCL10 and IL-33, and increased frequency of high IL-27 and CCL-11 responders when compared with patients infected with *S. mansoni* only.

### Multiple Regression Models

Table 6 presents the first multiple logistic regression model that describes the effect of ATL history and hematological and immunological parameters on active schistosomiasis in the study population. The analysis showed that previous ATL and high serum level of CCL3 (>5,740 pg/ml) and CCL17 (>245 pg/ml) were positively associated with *S. mansoni* infection. Furthermore, individuals with detectable serum levels of IL-17 had a six-fold higher chance to have active *S. mansoni* infection.

**Table 6:** Multiple regression model showing the association between *S. mansoni* infection with serum levels of immune mediators and previous report of ATL in individuals living in the rural community of Brejo do Amparo, Januária, Minas Gerais, Brazil.

***S. mansoni*** **infection** **+** **ATL history**^**a**^				
**Variable**	**Odds ratio**	**Z-score**	***p*****-value**	**CI 95%**
IL-27 (>388pg/ml)	3.10	2.06	0.040^*^	1.05–9.11
CXCL10 (>115pg/ml)	0.33	−2.04	0.042^*^	0.11–0.95
IL-17 detectable	0.08	−2.00	0.045^*^	0.01–0.94
IL-33 detectable	0.37	−1.66	0.098	0.11–1.19
CCL17 (>245pg/ml)	0.37	−1.74	0.082	0.12–1.13

a *Adjusted by age*.

* *Statistically significant (p < 0.05)*.

To better understand the effect of previous ATL on the immune response against *S. mansoni*, a second multiple logistic regression model was built using only patients infected with *S. mansoni* (SM and SM+ATL groups). The analysis demonstrated that CXCL10 and IL-17 serum response were negatively associated with detected *Schistosoma*-infected individuals that reported previous ATL (Table 7). Specifically, *S. mansoni*-infected individuals with detectable serum IL-17 or with high CXCL10 levels (>115 pg/mL) have their chances of presenting a history of ATL reduced by 92% and 67%, respectively. In contrast, this multiple regression model also showed that *S. mansoni*-infected individuals with high serum concentrations (>388pg/ml) of IL-27 are, on average, three times more likely to have reported previous ATL. Although the multiple logistic regression model showed no significant association for IL-33 and CCL17 response and previous ATL, these variables were maintained in the final model for a better fit and the model was adjusted by age (Table 7).

**Table 7:** Multiple regression model showing an association between previous report of ATL with serum levels of immune mediators among *S. mansoni*-infected individuals living in the rural community of Brejo do Amparo, Januária, Minas Gerais, Brazil.

***S. mansoni*** **infection**^**a**^				
**Variables**	**Odds ratio**	**Z-score**	***p*****-value**	**CI 95%**
ATL history	1.96	2.10	0.036^*^	1.04-3.68
IL-17 detectable	6.22	2.23	0.026^*^	1.24-31.1
CCL3 (>5,740pg/mL)	1.94	2.15	0.031^*^	1.06-3.57
CCL17 (>245pg/mL)	2.26	2.49	0.013^*^	1.19-4.30

a *Adjusted by age*.

* *Statistically significant (p < 0.05)*.

## Discussion

Leishmaniasis and schistosomiasis are chronic infectious diseases that have a great impact on human health status in tropical developing countries. Epidemiological studies indicate overlapping risk areas and elevated endemicities for both parasites (35, 39, 41, 43, 53). However, few studies have investigated the effect of co-infection on the immunological response and morbidity of human hosts. Experimental studies in mice infected with *L. major* and *S. mansoni* suggested that a Th2 predominant response induced by *S. mansoni* rendered macrophages incapable of controlling intracellular amastigotes (38). Moreover, *Leishmania*-induced lesions had a shorter incubation period and took longer to heal as a result of the co-infection (38, 54). In human populations, individuals infected with *L. braziliensis* and parasitized with different helminths (including *S. mansoni*) presented a compromised response to the treatment of cutaneous lesions (35, 39), which is in line with what was observed in mice (55). Nevertheless, to the best of our knowledge, no study has evaluated the impact of previous exposure to dermotropic *Leishmania* species on the outcome of the *S. mansoni* infection in humans. In the current study, we verified that individuals with an ATL history showed modulation of the systemic inflammatory response induced by the *S. mansoni* infection, with possible impacts on the host's susceptibility.

The study evaluated residents from a poor rural community in the Northern region of the state of Minas Gerais, where people live in constant contact with the etiological agents of intestinal schistosomiasis and ATL (40, 43). Previous research in this population using several parasitological techniques for the diagnosis of *S. mansoni* indicated a high prevalence of schistosomiasis, but the large majority of the infected individuals had a low parasite burden (<100 epg) and no severe pathology associated with the infection (40). Similar clinical profiles have been reported in many endemic areas of schistosomiasis worldwide, due to successive chemotherapeutic interventions (40, 56–58). In the same municipality and study area, the presence of tegumentary leishmaniasis has been well-documented (43), and our study confirmed and expanded on this information. The cutaneous form of leishmaniasis was predominant, and most individuals that reported ATL had a single lesion site. The predominance of cutaneous and localized ATL has also been reported in other studies carried out in Brazil (59–61). However, it is important to mention that most individuals with previous ATL that were treated at the CTPLJ (44% of the individuals who reported ATL in the study population) required more than 30 days of treatment for reepithelialization of *Leishmania*-induced cutaneous lesions and over 20% of these individuals had relapses/reinfections. Although it was not the focus of the current study, it is possible that the frequent exposure of individuals with tegumentary leishmaniasis to co-infection with *S. mansoni* has contributed to the longer period of anti-leishmaniasis treatment necessary for regression of the lesions, as previously observed by O'Neal et al. (35) and Unger et al. (39).

In the study population, *S. mansoni*-infected individuals showed a higher rate of eosinophilia, higher serum concentrations of CCL3 and CCL17, and a higher frequency of IL-17 responders in comparison with all the other groups of non-*Schistosoma*-infected individuals. The association of CCL17 and eosinophilia with *S. mansoni* infection was not unexpected, since these mediators are markers of a type-2 immune response, which is frequently reported in helminth-infected individuals (15, 16). Activated eosinophils are an important source of IL-4, IL-5, and IL-13 (62, 63), and this environment induces the production of CCL17 (64), which is described as a signature chemokine of helminth infection, especially *S. mansoni*, in humans (49, 65). Moreover, Jakubzick et al. (66) demonstrated that CCL17 production participates in CCR4^+^-cell recruitment to *S. mansoni*-induced lung granulomas, stimulating Th2 inflammatory markers, and increasing the hydroxyproline content in the tissue. Other studies have shown that CCL3 also participates in the polarization of Type-2 cytokine production and leukocyte recruitment to the liver of *Schistosoma*-infected mice (67). Moreover, CCL3 has been associated with fibrosis and hepatosplenic forms of human schistosomiasis (67–69). Even in patients infected with low parasite burden and without severe clinical symptoms, the serum concentration of CCL3 was positively associated with the intensity of the liver fibrosis (70). The correlation of CCL3 with hepatic fibrosis may be related to the fact that this chemokine stimulates the production of IL-13, which is considered a pro-fibrotic cytokine (71). The association of *Schistosoma* infection and production of IL-17 was initially established in experimental models, in which the cytokine production was an indicator of severe immunopathology and liver fibrosis (72, 73). In addition, the frequency of Th17 cells was positively associated with bladder pathology severity in the human populations infected with *S. haematobium* (74).

Our first multiple regression model confirmed the results from the univariable immunological analysis and showed a positive association of CCL17 and CCL3 higher responders and a high frequency of IL-17 responders with *S. mansoni* active infection in the study population. Interestingly, our data also showed that previous ATL was more frequent among individuals who were diagnosed with active schistosomiasis, and this association was confirmed in the multiple model, thus showing that individuals with an ATL history had a two-fold higher possibility of presenting active schistosomiasis. The data indicate that previous ATL may alter the host's susceptibility to *S. mansoni* infection in humans. However, among *Schistosoma*-infected individuals that reported previous ATL, the *S. mansoni* burden was always low (<100 epg).

Little is known about the duration of the host's immune response following specific treatment to tegumentary leishmaniasis and how it could interfere with the immune response to other infectious agents, such as *S. mansoni*. However, it was reported that patients infected with *L. braziliensis* presented positive PCR results years after the treatment, thus demonstrating that the parasite may remain in the skin, the bloodstream, or the lymphatic system for a long period after reepithelization of the lesion (75–77). Additionally, studies have shown that about 10% of patients in ATL-endemic areas had subclinical infections, for example, without cutaneous or mucosal lesions, despite being positive in the Montenegro's intradermal test (78). Therefore, we comparatively investigated the profile of the systemic immune response in individuals infected with *S. mansoni*, with or without a symptomatic ATL history. The data from the univariable analysis showed that *S. mansoni*-infected individuals who reported previous ATL had a lower rate of eosinophilia and a significant decrease in the frequency of CCL17 and IL-17 high-responders when compared with individuals infected with *S. mansoni* only, thus indicating a modulated immune response in these individuals. The second multiple regression model confirmed that previous ATL led to a modulatory immune profile in *S. mansoni*-infected individuals and showed a negative association with serum response of inflammatory mediators, including markers for Th1, Th2, and Th17 responses.

As observed herein, the modulation of both inflammatory and type-2 immune responses in the serum could be associated with the increased susceptibility to *S. mansoni* infection in individuals with an ATL history. The tendency for fewer IL-33 detection and CCL17 high responders in serum among the individuals from the SM+ATL group might be an indicative of a reduced type 2 immune response. A recent work (79) showed a negative association between plasma levels of IL-33, a type 2 immunity-related alarmin, and schistosomiasis and egg burden in school children from rural communities of Cameroon. Moreover, studies on population genetics with individuals living in endemic areas for schistosomiasis mansoni demonstrated that some polymorphisms in the SM1 genetic region, where several genes that code for type 2-immune cytokines, such as IL-3, IL-4, IL-5, IL-9, and IL-13, are located, are associated with susceptibility/resistance to schistosomiasis (80). Indeed, individuals with polymorphisms in IL-4 (−590T high IgE), IL-13 (−1055T high producer) and IFN-gamma (+874A high producer) demonstrated several correlations with resistance to reinfection (81, 82). Although the mechanisms behind this association are not yet fully understood, there is evidence that the SM1 genetic region (5q31-q33) is linked to a locus that regulates IgE levels (83), an antibody classically involved in protection against *Schistosoma* infection (84–87). Moreover, the multiple analysis demonstrated that an ATL history also promoted a significant modulation of serum CXCL10 and IL-17 responses in *Schistosoma*-infected individuals. The secretion of CXCL10 is directly associated with IFN-γ production, a hallmark of Th1 activation (88, 89). Experimental data obtained from mice immunized with radiation-attenuated schistosome larvae (90) and from mice infected with male *S. mansoni* parasites, but not female or mixed infection (91), indicated that the control of the parasite burden in the skin is associated with increased production of inflammatory chemokines and cytokines, especially CXCL10, CCL3, and IL-12. These mediators lead to neutrophil, eosinophil, and macrophage recruitment. Therefore, the modulation of CXCL10 serum response in *S. mansoni*-infected individuals with reports of ATL could also contribute to the increased susceptibility to this trematode infection.

The association between previous ATL and the modulation of the host's Th1/Th2 immune responses could also affect the *S. mansoni* egg production and/or elimination. As verified in our univariate analyses, none of the infected individuals with previous report of ATL eliminated more than 100 epg. Some studies in experimental models demonstrate that pro-inflammatory cytokines, such as TNF-α, stimulate the survival and fertility of *S. mansoni* adult worms (92, 93). Additionally, a type 2 immune response, with possible participation of tissue eosinophils, is also necessary to assist parasite egg elimination through the intestinal endothelium (94, 95). The modulation of inflammatory mediators detected in individuals with previous ATL and active schistosomiasis could affect these responses. However, only 10 individuals from the study population had a high *S. mansoni* burden, which did not allow a more detailed analysis.

In addition, it is important to notice that intense Th2/Th1/Th17 responses were also associated with the severity of the liver pathology in schistosomiasis (19, 72, 74, 96). Therefore, the modulation of Th1/Th17 inflammatory mediators in individuals with previous ATL history and active *S. mansoni* infection, as demonstrated by multiple regression analysis, could also interfere in the *S. mansoni*-induced pathology. Unfortunately, we were not able to better explore the possible effects of previous ATL on the morbidity caused by schistosomiasis in the study population because most of the infected individuals had a very low parasite burden, with no reported cases of severe schistosomiasis, known as the hepatosplenic form of the disease. Moreover, hemoglobin concentration and platelet count, which are classical parameters associated with schistosomiasis mansoni's morbidity (50, 97), did not differ significantly between groups. Further studies will be necessary to demonstrate whether the modulation of the inflammatory response by previous ATL can alter the morbidity of schistosomiasis.

The multiple analysis also showed that an ATL history was positively associated with high IL-27 response in *Schistosoma*-infected individuals. IL-27 is a cytokine produced by activated antigen-presenting cells (APCs), such as macrophages and dendritic cells (DCs) (98), and has an immunomodulatory role. Published data (99, 100) have suggested the modulatory effect of IL-27 on Th1 response and tissue lesion in subclinical infections with *L. braziliensis*. Also, mice deficient in the production of IL-27 (WSX-1^−/−^) and infected with *S. mansoni* produced lower levels of IFN-γ and less severe fibrosis, although these immune alterations did not significantly affect the severity of the disease (101). Therefore, the increased IL-27 response could downmodulate the CXCL10 and IL-17 responses observed in *S. mansoni*-infected individuals with a previous history of ATL. Another cytokine that has been associated with the modulation of the *Schistosoma*-induced immune response is IL-10. In the current study, few individuals had detectable levels of IL-10 in the serum and most of them were uninfected. The high limit of detection of IL-10 by the ELISA applied in the current study would be a limitation for the analysis. However, the significantly higher frequency of IL-10 responders in non-infected individuals could be a consequence of the constant exposure of the study population to chronic infectious diseases, such as schistosomiasis and leishmaniasis. IL-10 response among individuals with previous exposure to *S. mansoni* infection has been detected in other endemic areas (102, 103). Despite the lack of association between the IL-10 response and the modulatory profile of the immune response observed in *S. mansoni*-infected individuals with previous ATL, we could not rule out the possible participation of IL-10-mediated mechanisms in this process, since only serum samples were evaluated. It is possible that IL-10-producing cells may be recruited to egg-retention sites, such as the liver and the intestines.

Finally, although we were able to show some of the impacts that ATL may have on schistosomiasis, it is important to mention that the current data were derived from a cross-sectional study and, therefore, present some inherent limitations, such as the impediment of causal inferences. Besides, an important methodology limitation is the inability to determine when individuals were infected. Additionally, some previous cases of ATL could not be confirmed in the medical records, and subclinical individuals were not identified in this study. Regarding other parasitic infections, their prevalence was low in the study population and cases were evenly distributed among the groups. Another caveat worth mentioning is the absence of a more detailed evaluation of the symptoms of schistosomiasis in the study population, which limited the inferences regarding the *S. mansoni*-induced pathology. Therefore, complementary studies are necessary to further understand the modulatory effect of ATL on schistosomiasis.

In conclusion, we demonstrated that previous ATL is associated with reduced serum levels of inflammatory mediators induced by *S. mansoni* infection, and that such reduction was detected even in individuals infected with low parasite burden. This immune modulation may predispose these individuals to a higher susceptibility for the schistosome infection. On the other hand, the association of these diseases might also control intense inflammatory responses, which are normally associated with the more severe clinical cases of schistosomiasis.

## Data Availability Statement

The original contributions presented in the study are included in the article/Supplementary Material, further inquiries can be directed to the corresponding author/s.

## Ethics Statement

The studies involving human participants were reviewed and approved by Research Ethics Committee of Institute René Rachou/Fiocruz/MG (CAAE#21824513.9.0000.5091) Ethics Committee of the Federal University of Minas Gerais (CAAE: #69370517.3.0000.5149). Written informed consent to participate in this study was provided by the participants' legal guardian/next of kin.

## Author Contributions

GM, MC, SG, and DN-C: conceptualization. GM, SR, DC, GC, JS, VC, and SG: performed experiments. GM, SR, DC, GC, JS, VC, MC, and DN-C: data analysis. SG, MC, and DN-C: supervision. SG and DN-C: resources and project administration. GM and DN-C: wrote the manuscript. All authors reviewed and approved the manuscript.

## Conflict of Interest

The authors declare that the research was conducted in the absence of any commercial or financial relationships that could be construed as a potential conflict of interest.

## References

[1] Hotez PJ Kamath A. Neglected tropical diseases in subSaharan Africa: review of their prevalence, distribution, and disease burden. PLoS Neglected Trop Dis. (2009) 3:e412. 10.1371/journal.pntd.0000412 19707588 PMC2727001

[2] Weerakoon KG Gobert GN Cai P McManus DP. Advances in the Diagnosis of Human Schistosomiasis. Clin Microbiol Rev. (2015) 28:939–67. 10.1128/CMR.00137-14 26224883 PMC4548261

[3] World Health Organization. Global Health Estimates 2016: Deaths by Cause, Age, Sex, by Country and by Region, 2000–2016 (2018). Available online at: https://www.who.int/healthinfo/global_burden_disease/estimates/en/ (accessed October 31, 2020).

[4] Hiatt RA Ottesen EA Sotomayor ZR Lawley TJ. Serial observations of circulating imune complexes inpatientswith acute schistosomiasis. J Infect Dis. (1980) 142:665–70. 10.1093/infdis/142.5.665 7462683

[5] De Jesus AR Silva A Santana LB Magalhães A De Jesus AA De Almeida RP. Clinical and immunologic evaluation of 31 patients with acute schistosomiasis mansoni. J Infect Dis. (2002) 185:98–105. 10.1086/324668 11756987

[6] Katz N Almeida K. Esquistossomose, xistosa, barriga d'água. Ciência e Cultura. (2003) 55:38–43.

[7] Abath FGC Morais CN Montenegro CE Wynn TA Montenegro SM. Immunopathogenic mechanisms in schistosomiasis: what can be learnt from human studies? Trends Parasitol. (2006) 22:85–91. 10.1016/j.pt.2005.12.004 16380294

[8] Mazigo HD Nuwaha F Wilson S Kinung'hi SM Morona D Waihenya R. Epidemiology and interactions of Human Immunodeficiency Virus - 1 and *Schistosoma mansoni* in sub-Saharan Africa. Infect Dis Poverty. (2013) 2:2. 10.1186/2049-9957-2-2 23849678 PMC3707091

[9] Pearce EJ Macdonald AS. The immunobiology of schistosomiasis. Nat Rev Immunol. (2002) 2:499–511. 10.1038/nri843 12094224

[10] Stadecker MJ Hernandez HJ. The immune response and immunopathology in infection with *Schistosoma mansoni*: a key role of major egg antigen Sm-p40. Parasite Immunol. (1998) 20:217–21. 10.1046/j.1365-3024.1998.00150.x 9651922

[11] Dunne DW Pearce EJ. Immunology of hepatosplenic schistosomiasis mansoni: a human perspective. Microbes Infec. (1999) 1:533–60. 10.1016/s1286-4579(99)80095-1 10603572

[12] Grzych JM Pearce E Cheever A Caulada ZA Caspar P Heiny S. Egg deposition is the major stimulus for the production of Th2 cytokines in murine schistosomiasis mansoni. J Immunol. (1991) 146:1322–27. 1825109

[13] Pearce EJ Caspar P Grzych JM Lewis FA Sher A. Downregulation of Th1 Cytokine production accompanies induction of Th2 responses by a parasitic helminth, *Schistosoma mansoni*. J Exp Med. (1991) 173:159–66. 10.1084/jem.173.1.159 1824635 PMC2118762

[14] Helmy AH Abdel-Hady AA El-Shanawany F Hammam O Abdel-Hady A. The pharmacological approach to reserve portal hypertension and hepatic schistosomal fibrosis in Egypt, control experimental study. J Egypt Soc Parasitol. (2005) 35:731–50. 16333884

[15] Colley DG Secor WE. Immunology of human schistosomiasis. Parasite Immunol. (2014) 36:347–57. 10.1111/pim.12087 25142505 PMC4278558

[16] Araújo MI De Jesus AR Bacellar O. Evidence of a T helper type 2 activation in human schistosomiasis. Euro J Immunol. (1996) 26:1399–403. 10.1002/eji.1830260633 8647223

[17] Barron L Wynn TA. Fibrosis is regulated by Th2 and Th17 responses and by dynamic interactions between fibroblasts and macrophages. Am J Physiol Gastrointest Liver Physiol. (2011) 300:723–8. 10.1152/ajpgi.00414.2010 21292997 PMC3302189

[18] Lenzi HL Kimmel E Schechtman H Pelajo-Machado M Romanha WS Pacheco RG. Histoarchitecture of schistosomal granuloma development and involution: morphogenetic and biomechanical approaches. Mem Inst Oswaldo Cruz. (1998) 93:141–51. 10.1590/s0074-02761998000700020 9921336

[19] Cheever AW Hoffmann KF Wynn TA. Immunopathology of schistosomiasis mansoni in mice and men. Immunol Today. (2000) 21:465–66. 10.1016/s0167-5699(00)01626-1 10953099

[20] Hams E Aviello G Fallon PG. The *Schistosoma* granuloma: friend or foe? Front Immunol. (2013) 4:89. 10.3389/fimmu.2013.00089 23596444 PMC3625856

[21] Hesse M Piccirillo CA Belkaid Y Prufer J Mentink-Kane M Leusink M. The pathogenesis of schistosomiasis is controlled by cooperating IL-10- producing innate effector and regulatory T cells. J Immunol. (2004) 172:3157–66. 10.4049/jimmunol.172.5.3157 14978122

[22] Lundy SK Lukacs NW. Chronic schistosome infection leads to modulation of granuloma formation and systemic immune suppression. Front Immunol. (2013) 4:39. 10.3389/fimmu.2013.00039 23429492 PMC3576626

[23] Alvar J Vélez ID Bern C Herrero M Desjeux P Cano J. Leishmaniasis worldwide and global estimates of its incidence. PLos ONE. (2012) 7:1–12. 10.1371/journal.pone.0035671 22693548 PMC3365071

[24] Liew FY Millott S Parkinson C Palmer RM Moncada S. Macrophage killing of *Leishmania* parasite *in vivo* is mediated by nitric oxide from L-arginine. J Immunol. (1990) 144:4794–97. 2351828

[25] Sacks D Noben-Trauth N. The immunology of susceptibility and resistance to *Leishmania major* in mice. Nat Rev Immunol. (2002) 2:845–58. 10.1038/nri933 12415308

[26] Von Stebut E Ehrchen JM Belkaid Y Kostka SL Mölle K Knop J. Interleukin 1α promotes Th (1) differentiation and inhibits disease progression in *Leishmania* major–susceptible BALB/c mice. J Exp Med. (2003) 198:191–99. 10.1084/jem.20030159 12860932 PMC2194079

[27] Mukbel RM Jr CP Gibson K Ghosh M Petersen C Jones DE. Macrophage killing of *Leishmania amazonensis* amastigotes requires both nitric oxide and superoxide. Am J Trop Med Hyg. (2007) 76:669–75. 10.4269/ajtmh.2007.76.669 17426168

[28] Castellano LR Filho DC Argiro L Dessein H Prata A Dessein A. Th1/Th2 immune responses are associated with active cutaneous leishmaniasis and clinical cure is associated with strong interferon-γ production. Hum Immunol. (2009) 70:383–90. 10.1016/j.humimm.2009.01.007 19480861

[29] Maspi N Abdoli A Ghaffarifar F. Pro-and anti-inflammatory cytokines in cutaneous leishmaniasis: a review. Pathog Glob Health. (2016) 110:247–60. 10.1080/20477724.2016.1232042 27660895 PMC5070640

[30] Nylén S Eidsmo L. Tissue damage and immunity in cutaneous leishmaniasis. Parasite Immunol. (2012) 34:51–61. 10.1111/pim.12007 23009296

[31] Pasparakis M Haase I Nestle FO. Mechanisms regulating skin immunity and inflammation. Nat Rev Immunol. (2014) 14:289–301. 10.1038/nri3646 24722477

[32] Campos TM Novais FO Saldanha M Costa R Lordelo M Celestino D. Granzyme B Produced by Natural Killer Cells Enhances Inflammatory Response and Contributes to the Immunopathology of Cutaneous Leishmaniasis. J Infect Dis. (2020) 221:973–82. 10.1093/infdis/jiz538 31748808 PMC7050991

[33] Allen JE Wynn TA. Evolution of Th2 Immunity: a rapid repair response to tissue destructive pathogens. PLoS Pathog. (2011) 7:e1002003. 10.1371/journal.ppat.1002003 21589896 PMC3093361

[34] Butterworth AE Curry AJ Dunne DW Fulford AJ Kimani G Kariuki HC. Immunity and morbidity in human schistosomiasis mansoni. Trop Geogr Med. (1994) 46:197–208. 7825222

[35] O'Neal SE Guimarães LH Machado PR Alcântara L Morgan DJ Passos S. Influence of helminth infections on the clinical course of and immune response to *Leishmania braziliensis* cutaneous leishmaniasis. J Infect Dis. (2007) 195:142–8. 10.1086/509808 17152018

[36] Azeredo-Coutinho RBG Pimentel MI Zanini GM Madeira MF Cataldo JI Schubach AO. Intestinal helminth coinfection is associated with mucosal lesions and poor response to therapy in American tegumentary leishmaniasis. Acta Trop. (2016) 15:42–9. 10.1016/j.actatropica.2015.10.015 26519200

[37] Gregorio AW Vasconcellos MRA Enokihara MMSS Guerra JM Nonogaki S Tomimori J. Cutaneous schistosomiasis and leishmaniasis coinfection: a case report. J Eur Acad Dermatol Venereol. (2018) 33:1781–3. 10.1111/jdv.15521 30801816

[38] La Flamme AC Scott P Pearce E. Schistosomiasis delays lesion resolution during *Leishmania major* infection by imparing parasite killing by macrophages. Parasite Immunol. (2002) 24:339–45. 10.1046/j.1365-3024.2002.00473.x 12164819

[39] Unger A O'Neal S Machado PRL Guimarães LH Morgan DJ Schriefer A. Association of treatment of American cutaneous leishmaniasis prior to ulcer development with high rate of failure in Northeastern Brazil. Am J Trop Med Hyg. (2009) 80:574–9. 10.4269/ajtmh.2009.80.574 19346378 PMC3557504

[40] Oliveira WJ Magalhães FDC Elias AMS de Castro VN Favero V Lindholz CG. Evaluation of diagnostic methods for the detection of intestinal schistosomiasis in endemic areas with low parasite loads: Saline gradient, Helmintex, Kato-Katz and rapid urine test. PLoS Negl Trop Dis. (2018) 12:e0006232. 10.1371/journal.pntd.0006232 29470516 PMC5823366

[41] Resende SD Magalhães FC Rodrigues-Oliveira JL Castro VN Souza CS Oliveira. Modulation of allergic reactivity in humans is dependent on *Schistosoma mansoni* parasite burden, low levels of IL-33 or TNF-α and high levels of IL-10 in serum. Front Immunol. (2019) 9:3158. 10.3389/fimmu.2018.03158 30713536 PMC6345678

[42] Magalhães F Resende S Senra C Graeff-Teixeira C Enk M Coelho P. Accuracy of real-time polymerase chain reaction to detect *Schistosoma mansoni* – infected individuals from an endemic area with low parasite loads. Parasitol. (2020) 147:1140–8. 10.1017/S003118202000089X 32484122 PMC10317740

[43] Cardoso DT de Souza DC de Castro VN Geiger SM Barbosa DS. Identification of priority areas for surveillance of cutaneous leishmaniasis using spatial analysis approaches in Southeastern Brazil. BMC Infect Dis. (2019) 19:318. 10.1186/s12879-019-3940-4 30975100 PMC6458754

[44] Hoffman WA Pons JA Janer JL. The Sedimentation-Concentration Method in Schistosomiasis mansoni. PR Health Sci J. (1934) 9:283–91.

[45] Kato K Miura M. Comparative examinations. Japan J Parasitol. (1954) 3:5.

[46] Katz N Chaves A Pellegrino J. A simple device for quantitative stool thick- smear technique in Schistosomiasis mansoni. Rev Inst Med Trop São Paulo. (1972) 14:399–400. 4675644

[47] Coelho PMZ Jurberg AD Oliveira ÁA Katz N. Use of a saline gradient for the diagnosis of schistosomiasis. Mem Inst Oswaldo Cruz. (2009) 104:720–3. 10.1590/s0074-02762009000500010 19820832

[48] Teixeira CF Neuhauss E Ben R Romanzini J Graeff-Teixeira C. Detection of *Schistosoma mansoni* eggs in feces through their interaction with paramagnetic beads in a magnetic field. Plos Negl Trop Dis. (2007) 1:e73. 10.1371/journal.pntd.0000073 18060086 PMC2100366

[49] Castro VN Rodrigues JL Cardoso DT Resende SD Magalhães FC Souza DC. Systemic Cytokine and Chemokine Profiles in Individuals with *Schistosoma mansoni* Infection and Low Parasite Burden. Front Immunol. (2018) 9:2975. 10.3389/fimmu.2018.02975 30619332 PMC6305627

[50] Lambertucci JR dos Santos Silva LC Antunes CM. Aspartate aminotransferase to platelet ratio index and blood platelet count are good markers for fibrosis evaluation in schistosomiasis mansoni. Rev Soc Bras Med Trop. (2007) 40:599. 10.1590/s0037-86822007000500023 17992423

[51] Rosenfeld LG Malta DC Szwarcwald CL Bacal NS Cuder MAM Pereira CA. Reference values for blood count laboratory tests in the Brazilian adult population, National Health Survey. Rev Bras de Epid. (2019) 22:E190003-SUPL. 10.1590/1980-549720190003.supl.2 31596374

[52] World Health Organization. Helminth control in school-age children: A guide for managers of control programmes. Geneva, 2002. Available online at: https://www.who.int/neglected_diseases/resources/9789241548267/en/ (accessed October 31, 2020).

[53] Scholte RG Gosoniu L Malone JB Chammartin F Utzinger J Vounatsou P. Predictive risk mapping of schistosomiasis in Brazil using Bayesian geostatistical models. Acta Trop. (2014) 132:57–63. 10.1016/j.actatropica.2013.12.007 24361640

[54] Coelho PMZ Mayrink W Dias M Pereira LH. Susceptibility to *Leishmania mexicana* of mice infected with *Schistosoma mansoni*. T Roy Soc Trop Med H. (1980) 74:141. 7434414 10.1016/0035-9203(80)90038-3

[55] Khayeka–Wandabwa C Kutima HL Nyambati VCS Ingonga J Oyoo–Okoth E Karani LW. Combination therapy using Pentostam and Praziquantel improves lesion healing and parasite resolution in BALB/c mice co-infected with *Leishmania major* and *Schistosoma mansoni*. Parasit. Vectors. (2013) 6:244. 10.1186/1756-3305-6-244 PMC3765423 23968249

[56] Hofstede SN Tami A van Liere GA Ballén D Incani RN. Long-term effect of mass chemotherapy, transmission and risk factors for *Schistosoma mansoni* infection in very low endemic communities of Venezuela. Acta Trop. (2014) 140:68–76. 10.1016/j.actatropica.2014.08.003 25128702

[57] Krauth SJ Greter H Stete K Coulibaly JT Traoré SI Ngandolo BN. All that is blood is not schistosomiasis: experiences with reagent strip testing for urogenital schistosomiasis with special consideration to very-low prevalence settings. Parasit Vectors. (2015) 10:584. 10.1186/s13071-015-1165-y 26554822 PMC4641389

[58] He P Gordon CA Williams GM Li Y Wang Y Hu J. Real-time PCR diagnosis of *Schistosoma japonicum* in low transmission areas of China. Infect Dis Poverty. (2018) 7:8 10.1186/s40249-018-0390-y 29394958 PMC5796516

[59] Lima Filho JHC Steindel M. Aspectos clínicos e epidemiológicos da leishmaniose cutânea no Estado de Santa Catarina. Arq Catarinenses Med. (1998) 27:25–31.

[60] Guerra JAO Ribeiro JAS Coelho LIARC Barbosa MGV Paes MG. Epidemiologia da leishmaniose tegumentar na comunidade São João, Manaus, Amazonas, Brasil. Caderno de Saúde Pública. (2006) 22:2319–27. 10.1590/S0102-311X2006001100006 17091169

[61] Da Silva PLN Versiani CMC Chagas RB Da Rocha RG Majuste R Da Silva JS. Estudo da leishmaniose tegumentar americana na cidade de Montes Claros/MG: aspectos epidemiológico, clínico e terapêutico. J H Sci Inst. (2014) 32:38–42.

[62] Davies SJ Smith SJ Lim KC Zhang H Purchio AF McKerrow JH. *In vivo* imaging of tissue eosinophilia and eosinopoietic responses to schistosome worms and eggs. Int J Parasitol. (2005) 35:851–9. 10.1016/j.ijpara.2005.02.017 15950229 PMC2891237

[63] Rothenberg ME Hogan SP. The eosinophil. Annu Rev Immunol. (2006) 24:147–74. 10.1146/annurev.immunol.24.021605.090720 16551246

[64] Sekiya T Yamada H Yamaguchi M Yamamoto K Ishii A Yoshie O.: Increased levels of a TH2-type CC chemokine thymus and activation-regulated chemokines (TARC) in serum and induced sputum of asthmatics. Allergy. (2002) 57:173–7. 10.1034/j.1398-9995.2002.5720256.x 11929424

[65] Geiger SM Jardim-Botelho A Williams W Alexander N Diemert DJ Bethony JM. Serum CCL 11 (eotaxin-1) and CCL 17 (TARC) are serological indicators of multiple helminth infections and are driven by *Schistosoma mansoni* infection in humans. Trop Med Int Health. (2013) 18:750–60. 10.1111/tmi.12095 23496801

[66] Jakubzick C Wen H Matsukawa A Keller M Kunkel SL Hogaboam CM. Role of CCR4 ligands, CCL17 and CCL22, during *Schistosoma mansoni* egg-induced pulmonary granuloma formation in mice. Am J Pathol. (2004) 165:1211–21. 10.1016/S0002-9440(10)63381-0 15466387 PMC1618636

[67] Souza AL Roffe E Pinho V Souza DG Silva AF Russo RC. Potential role of the chemokines macrophage inflammatory protein-1alpha in human and experimental schistosomiasis. Infect Immun. (2005) 73:2515–23. 10.1128/IAI.73.4.2515-2523.2005 15784598 PMC1087406

[68] Falcão PL Correa-Oliveira R Fraga LA Tavani A Proudffot AEI Wells TNC. Plasma concentrations and role of macrophage inflammatory protein-1alpha during chronic *Schistosoma mansoni* infection in humans. J Infect Dis. (2002) 186:1696–700. 10.1086/345370 12447751

[69] Sousa-Pereira SR Teixeira AL Silva LC Souza AL Antunes CM Teixeira MM. Serum and cerebral spinal fluid levels of chemokines and Th2 cytokines in *Schistosoma mansoni* myeloradiculopathy. Parasite Immunol. (2006) 28:473–8. 10.1111/j.1365-3024.2006.00896.x 16916371

[70] Teixeira MM Lambertucci JR Antunes CMF Carneiro M Negrão-Corrêa D. Plasma levels of innate immune mediators are associated with liver fibrosis in low parasite burden *Schistosoma mansoni*-infected individuals. Scand J Immunol. (2018) 87:e12642. 10.1111/sji.12642 29363152

[71] Alves Oliveira LF Moreno EC Gazzinelli G Martins-Filho OA Silveira AMS Gazzinelli A. Cytokine production associated with periportal fibrosis during chronic Schistosomiasis mansoni in humans. Infect Immun. (2006) 74:1215–21. 10.1128/IAI.74.2.1215-1221.2006 16428771 PMC1360316

[72] Rutitzky LI Stadecker MJ. Exacerbated egg-induced immunopathology in murine *Schistosoma mansoni* infection is primarily mediated by IL-17 and restrained by IFN-γ. Eur J Immunol. (2011) 41:2677–87. 10.1002/eji.201041327 21660933 PMC3679923

[73] Wang B Liang S Wang Y Zhu XQ Gong W Zhang HQ. Th17 Down-regulation Is Involved in Reduced Progression of Schistosomiasis Fibrosis in ICOSL KO Mice. PLoS Negl Trop Dis. (2015) 9:e0003434. 10.1371/journal.pntd.0003434 25590646 PMC4295877

[74] Mbow M Larkin BM Meurs L Wammes LJ de Jong SE Labuda LA. 2013. T-helper 17 cells are associated with pathology in human schistosomiasis. J Infect Dis. (2013) 207:186. 10.1093/infdis/jis654 23087431 PMC3571236

[75] Guevara P Ramirez JL Rojas E Scorza JV Gonzalez N Anez N. *Leishmania braziliensis* in blood 30 years after cure. Lancet. (1993) 341:1341. 10.1016/0140-6736(93)90845-8 8098463

[76] Schubach A Haddad F Neto MPO Degrave W Pirmez C Grimaldi G Jr. Detection of Leishmania DNA by polymerase chain reaction in scars of treated human patients. J Infec Dis. (1998) 178:911–14. 10.1086/515355 9728572

[77] Haddad F Schubach A Oliveira-Neto MP Degrave W Pirmez C Fernandes O. Detection of minicircle DNA of *Leishmania* in paraffin-embedded tissue from scars of treated patients. Mem Inst Oswaldo Cruz. (1996) 91(Suppl):185.

[78] Follador I Araujo C Bacellar O Araujo CB Carvalho LP Almeida RP. Epidemiologic and immunologic findings for the subclinical form of *Leishmania braziliensis* infection. Clin Infect Dis. (2002) 34:E54–E58. 10.1086/340261 12015707

[79] Kamdem SD Konhawa F Kuemkon EM Meyo Kamguia L Tchanana GK Nche F. Negative Association of Interleukin-33 Plasma Levels and Schistosomiasis Infection in a Site of Polyparasitism in Rural Cameroon. Front Immunol. (2019) 10:2827. 10.3389/fimmu.2019.02827 31849991 PMC6901687

[80] Marquet S Abel L Hillaire D Dessein H Kalil J Feingold J. Genetic localization of a locus controlling the intensity of infection by *Schistosoma mansoni* on chromosome 5q31-q33. Nat Genet. (1996) 14:181–4. 10.1038/ng1096-181 8841190

[81] Gatlin MR Black CL Mwinzi PN Secor WE Karanja DM Colley DG. Association of the gene polymorphisms IFN-gamma +874, IL-13−1055 and IL-4−590 with patterns of reinfection with *Schistosoma mansoni*. PLoS Negl Trop Dis. (2009) 3:e375. 10.1371/journal.pntd.0000375 19190772 PMC2631135

[82] Grant AV Araujo MI Ponte EV Oliveira RR Gao P Cruz AA. Functional polymorphisms in IL13 are protective against high *Schistosoma mansoni* infection intensity in a Brazilian population. PLoS ONE. (2012) 7:e35863. 10.1371/journal.pone.0035863 22574126 PMC3345031

[83] Marsh DG Neely JD Breazeale DR Ghosh B Freidhoff LR Ehrlich-Kautzky E. Linkage analysis of IL4 and other chromosome 5q31.1 markers and total serum immunoglobulin E concentrations. Science. (1994) 264:1152–6. 10.1126/science.8178175 8178175

[84] Hagan P Blumenthal UJ Dunn D Simpson AJ Wilkins HA. Human IgE, IgG4 and resistance to reinfection with Schistosoma haematobium. Nature. (1991) 349:243–45. 10.1038/349243a0 1898985

[85] De Andres B Rakasz E Hagen M McCormik ML Mueller AL Elliot D. Lack of Fc-epsilon receptors on murine eosinophils: implications for the functional significance of elevated IgE and eosinophils in parasitic infections. Blood. (1997) 89:3826–36. 9160690

[86] Dunne DW Butterworth AE Fulford AJ Kariuki HC Langley JG Ouma JH. Immunity after treatment of human schistosomiasis: association between IgE antibodies to adult worm antigens and resistance to reinfection. Eur J Immunol. (1992) 22:1483–94. 10.1002/eji.1830220622 1601036

[87] Negrão-Corrêa D Fittipaldi JF Lambertucci JR Teixeira MM Antunes CMF Carneiro M. Association of Schistosoma mansoni-Specific IgG and IgE Antibody Production and Clinical Schistosomiasis Status in a Rural Area of Minas Gerais, Brazil. PLoS ONE. (2014) 9:e88042. 10.1371/journal.pone.0088042 24505371 PMC3913716

[88] Antonelli A Ferrari SM Giuggioli D Ferrannini E Ferri C Fallahi P. Chemokine (C–X–C motif) ligand (CXCL) 10 in autoimmune diseases. Autoimmun Rev. (2014) 13:272–80. 10.1016/j.autrev.2013.10.010 24189283

[89] Chalin A Lefevre B Devisme C Pronier C Carrière V Thibault V. Serum CXCL10, CXCL11, CXCL12, and CXCL14 chemokine patterns in patients with acute liver injury. Cytokine. (2018) 111:500–4. 10.1016/j.cyto.2018.05.029 29880273

[90] Hogg KG Kumkate S Anderson S Mountford AP. Interleukin-12 p40 secretion by cutaneous CD11c+ and F4/80+ cells is a major feature of the innate immune response in mice that develop Th1-mediated protective immunity to *Schistosoma mansoni*. Infec Immun. (2003) 71:3563–71. 10.1128/iai.71.6.3563-71.2003 12761141 PMC155763

[91] Sombetzki M Koslowski N Rabes A Seneberg S Winkelmann F Fritzsche C. Host defense versus immunosuppression: unisexual infection with male or female *Schistosoma mansoni* differentially impacts the immune response against invading cercariae. Front Immunol. (2018) 9:861. 10.3389/fimmu.2018.00861 29743881 PMC5930291

[92] Amiri P Locksley RM Parslow TG Sadick M Rector E Ritter D. Tumour necrosis factor alpha restores granulomas and induces parasite egg-laying in schistosome-infected SCID mice. Nature. (1992) 356:604–7. 10.1038/356604a0 1560843

[93] Davies SJ Lim KC Blank RB Kim JH Lucas KD Hernandez DC. Involvement of TNF in limiting liver pathology and promoting parasite survival during schistosome infection. Int J Parasitol. (2004) 34:27–36. 10.1016/j.ijpara.2003.10.010 14711587 PMC2859728

[94] Lenzi HL Pacheco RG Pelajo-Machado M Panasco MS Romanha WS Lenzi JA. Immunological system and *Schistosoma mansoni*: co-evolutionary immunobiology. What is the eosinophil role in parasite-host relationship? Mem Inst Oswaldo Cruz. (1997) 92:19–32. 10.1590/S0074-02761997000800005 9698912

[95] Schwartz C Fallon PG. *Schistosoma* “Eggs-Iting” the Host: Granuloma Formation and Egg Excretion. Front Immunol. (2018) 9:2492. 10.3389/fimmu.2018.02492 30459767 PMC6232930

[96] Liang YJ Luo J Lu Q Zhou Y Wu HW Zheng D. Gene profile of chemokines on hepatic stellate cells of schistosome-infected mice and antifibrotic roles of CXCL9/10 on liver non-parenchymal cells. PLoS ONE. (2012) 7:e42490. 10.1371/journal.pone.0042490 22905138 PMC3414521

[97] Friedman JF Kanzaria HK McGarvey ST. Human schistosomiasis and anemia: the relationship and potential mechanisms. Trends Parasitol. (2005) 21:386–92. 10.1016/j.pt.2005.06.006 15967725

[98] Kastelein RA Hunter CA Cua DJ. Discovery and biology of IL-23 and IL-27: related but functionally distinct regulators of inflammation. Annu Rev Immunol. (2007) 25:221–42. 10.1146/annurev.immunol.22.012703.104758 17291186

[99] Bittar RC Nogueira RS Vieira-Goncalves R Pinho-Ribeiro V Mattos MS Oliveira-Neto MP. T-cell responses associated with resistance to *Leishmania* infection in individuals from endemic areas for *Leishmania (Viannia) braziliensis*. Mem Inst Oswaldo Cruz. (2007) 102:625–30. 10.1590/s0074-02762007005000069 17710308

[100] Novoa R Bacellar O Nascimento M Cardoso TM Ramasawmy R Oliveira WN. IL-17 and Regulatory Cytokines (IL-10 and IL-27) in *L. braziliensis* Infection. Parasite Immunol. (2011) 33:132–6. 10.1111/j.1365-3024.2010.01256.x 21226726 PMC3077537

[101] Shainheit MG Saraceno R Bazzone LE Rutitzky LI Stadecker MJ. Disruption of interleukin-27 signaling results in impaired gamma interferon production but does not significantly affect immunopathology in murine schistosome infection. Infec Immun. (2007) 75:3169–77. 10.1128/IAI.01053-06 17403877 PMC1932859

[102] Malaquias LCC Falcao PL Silveira AMS Gazzinelli G Prata A Coffman RL. Cytokine regulation of human immune response to *Schistosoma mansoni*: analysis of the role of IL-4, IL-5 and IL-10 on peripheral blood mononuclear cell responses. Scand J Immun. (1997) 46:393–8. 10.1046/j.1365-3083.1997.d01-136.x 9350291

[103] Eltayeb NM Mukhtar MM Mohamed AB. Epidemiology of schistosomiasis in Gezira area Central Sudan and analysis of cytokine profiles. Asian Pac J Trop Med. (2013) 6:119–25. 10.1016/S1995-7645(13)60006-1 23339913

