# Supplementary material for: Previous History of American Tegumentary Leishmaniasis Alters Susceptibility and Immune Response Against Schistosoma mansoni Infection in Humans
Source: Front Immunol. 2021 Mar 11;12:630934. doi: 10.3389/fimmu.2021.630934 (PMC7990892; doi:10.3389/fimmu.2021.630934)
Supplement: Supplementary file 1 [file Table_1.docx]

**Supplementary Tables**

**Table S1.** Sociodemographic and parasitological profile of individuals living in the rural community of Brejo do Amparo, Januária, Minas Gerais, Brazil.

| **Gender^a^** | **n (%)** |
| --- | --- |
| Men | 122 (46.7) |
| Women | 135 (53.3) |
| **Education level^b^** | **n (%)** |
| No education | 123 (59.4) |
| Primary school | 33 (15.9) |
| Secondary school | 43 (20.8) |
| Higher education | 8 (3.9) |
| **Income^c^** | **n (%)** |
| <1 minimum wage | 19 (35.9) |
| 1–2 minimum wages | 16 (30.2) |
| >2 minimum wages | 18 (34.0) |
| **Protozoa parasites^a^** | **n (%)** |
| *Entamoeba coli* | 25 (10.0) |
| *Endolimax nana* | 22 (9.0.) |
| *Blastocystis* sp. | 11 (40) |
| *Ent. histolytica/dispar* | 09 (3.5) |
| *Giardia lamblia* | 04 (1.5) |
| *Iodamoeba sp.* | 02 (0.8) |
| *E. hartmanni* | 01 (0.4) |
| **Helminths parasites^a^** | **n (%)** |
| *Schistosoma mansoni* | 118 (46.0) |
| Hookworm | 23 (9.0) |
| *Enterobius vermicularis* | 6 (2.0) |
| *Trichuris trichiura* | 1 (0.4) |
| *Strongyloides stercoralis* | 1 (0.4) |

^a^Variables evaluated based in total of study population (257 residents).

^b^Variable evaluated in the individual questionnaire excluding children under 6 years of age (207 residents).

^c^Variables evaluated in the family questionnaire (53 residents).
