# Supplementary material for: Previous History of American Tegumentary Leishmaniasis Alters Susceptibility and Immune Response Against Schistosoma mansoni Infection in Humans
Source: Front Immunol. 2021 Mar 11;12:630934. doi: 10.3389/fimmu.2021.630934 (PMC7990892; doi:10.3389/fimmu.2021.630934)
Supplement: Supplementary file 2 [file Table_2.docx]

**Supplementary Table**

**Table S2.** Characteristics of *Schistosoma mansoni* infection in individuals living in the rural community of Brejo do Amparo, Januária, Minas Gerais, Brazil.

| ***S. mansoni* infection** | | |
| --- | --- | --- |
| **Gender** | **n (%)** | **p^a^** |
| Man | 55 (47.0) | 0.406 |
|  |  |  |
| Woman | 63 (53.0) |  |
| **Age** | **n (%)** | **p^a^** |
| 0-10 years | 13 (11.0) | 0.289 |
| 11-20 years | 26 (22.0) |  |
| 21-40 years | 33 (28.0) |  |
| 41-60 years | 32 (27.0) |  |
| > 60 years | 14 (12.0) |  |
| **General burden** | **n (%)** |  |
| < 12 epg | 77 (65.2) |  |
| 12–99 epg | 31 (26.3) |  |
| > 99 epg | 10 (8.5) |  |
| **Burden per gender (epg)** | **Md (IR 25 – 75%)** | **p^b^** |
| Man | 4 (2-33) | 0.413 |
| Woman | 4 (2-13) |  |
| **Buden per age (epg)** | **Md (IR 25 – 75%)** | **p^c^** |
| 0-10 years | 2 (2-22) | 0.110 |
| 11-20 years | 4 (2-26) |  |
| 21-40 years | 12 (2-42) |  |
| 41-60 years | 3 (2-11) |  |
| > 60 years | 4 (2-24) |  |

M_d_ = median; IR = interquartile range; epg = eggs per gram of feces. p-value obtained by chi-square test (^a^), Mann-Whitney test (^b^), and Kruskal-Wallis, followed by Dunn's multiple comparisons post-test (^c^).
